# A Comparison of Epileptogenic Effect of Status Epilepticus Treated With Diazepam, Midazolam, and Pentobarbital in the Mouse Pilocarpine Model of Epilepsy

**DOI:** 10.3389/fneur.2022.821917

**Published:** 2022-05-20

**Authors:** Xiangzhen Tong, Zizhu Zhang, Jianping Zhu, Shuji Li, Shaogang Qu, Bing Qin, Yanwu Guo, Rongqing Chen

**Affiliations:** ^1^Guangdong Province Key Laboratory of Psychiatric Disorders, Department of Neurobiology, School of Basic Medical Sciences, Southern Medical University, Guangzhou, China; ^2^Department of Neurology, Nanfang Hospital, Southern Medical University, Guangzhou, China; ^3^Epilepsy Center and Department of Neurosurgery, The First Affiliated Hospital, Jinan University, Guangzhou, China; ^4^Guangdong Provincial Key Laboratory on Brain Function Repair and Regeneration, Department of Neurosurgery, The National Key Clinic Specialty, The Engineering Technology Research Center of Education Ministry of China, Zhujiang Hospital, Southern Medical University, Guangzhou, China; ^5^Key Laboratory of Mental Health of the Ministry of Education, Guangdong-Hong Kong-Macao Greater Bay Area Center for Brain Science and Brain-Inspired Intelligence, Southern Medical University, Guangzhou, China

**Keywords:** epilepsy, epileptogenesis, status epilepticus (SE), midazolam, diazepam, pentobarbital

## Abstract

Status epilepticus (SE) is a medical emergency associated with acute severe systemic damage and high mortality. Moreover, symptomatic SE is one of the highest risk factors for epileptogenesis. While the antiepileptic drugs (AEDs) are chosen in favor of acute control of SE, the potential short-term and long-term effects of such AEDs have been ignored in clinics. In this study, we hypothesized that AEDs that are used to control acute SE might affect the feasibility for the chronic development of epileptogenesis after SE. Therefore, we sought to compare the epileptogenic effects of SE that are terminated by three AEDs, i.e., diazepam, midazolam, and pentobarbital, which are widely used as first-line anti-SE AEDs. For this purpose, we used a mouse model of SE induced by intraperitoneal (i.p.) injection of lithium chloride (LiCl)-pilocarpine. The pilocarpine-induced SE was terminated with diazepam, midazolam, or pentobarbital. Then we compared short-term and long-term effects of SE with different AED treatments by examining SE-associated mortality and behavioral spontaneous recurrent seizures (SRSs) and by using magnetic resonance imaging (MRI) and immunohistochemistry to evaluate pathological and cellular alterations of mice in the different treatment groups. We found that i.p. injections of diazepam (5 mg/kg), midazolam (10 mg/kg), and pentobarbital (37.5 mg/kg) were able to terminate acute pilocarpine-SE effectively, while pentobarbital treatment showed less neuroprotective action against lethality in the short phase following SE. Long-term evaluation following SE revealed that SE treated with midazolam had resulted in relatively less behavioral SRS, less hippocampal atrophy, and milder neuronal loss and gliosis. Our data revealed an obvious advantage of midazolam vs. diazepam or pentobarbital in protecting the brain from epileptogenesis. Therefore, if midazolam provides as strong action to quench SE as other AEDs in clinics, midazolam should be the first choice of anti-SE AEDs as it provides additional benefits against epileptogenesis.

## Introduction

Epileptogenesis refers to the pathological and pathophysiological processes, which are initiated by initial brain insults and chronically results in the development of spontaneous recurrent seizures (SRSs). The time window between the initial brain insults and the first SRS is usually called the latent period. An initiating insult can be one of the wide ranges of brain damage that include neurological infections, traumatic brain injury (TBI), ischemia, intracerebral hemorrhage, intracranial tumor, unprovoked seizures, etc. During the latent period and throughout the whole process of epileptogenesis following the initial brain insults, comprehensive changes, e.g., neuronal loss, loss of balance of neuronal excitation and inhibition, gliosis, brain tissue sclerosis, brain structural and network reorganization, chronically occur in the brain regions that are vulnerable to epileptogenesis (1, 2). It is believed that the accumulation of those pathological and pathophysiological changes after initial brain insults gives rise to the formation of the epileptic brain and the occurrence of unprovoked SRS (1, 2).

When a seizure lasts for more than 5 min or two or more seizures occur too close to allow full recovery of epileptic activities between seizures, there is a medical emergency that is called status epilepticus (SE). In practical conditions, any SE should be treated as quickly as possible because poorly controlled SE is associated with severe systemic damage and high mortality. The treatments against SE are firstly aimed to terminate seizures with antiepileptic drugs (AEDs) and then to prevent acute seizure recurrence. Diazepam, midazolam, lorazepam, phenobarbital, phenytoin, and valproate are some of the widely used AEDs for the treatment of SE (3, 4). The treatment option is usually decided according to the stages and time points of SE. For example, diazepam, lorazepam, pentobarbital, and midazolam are recommended as the first-line choices against early convulsive SE, while phenytoin and valproate are considered to be second-line choices against established convulsive SE (3, 4). Among the numerous initial brain insults, symptomatic SE is one of the highest risk factors of epileptogenesis. Approximately 1.5–17% of patients develop SRS within 30 years after TBI depending on injury severity (5), 2.4–22% of patients develop SRS within 20 years after infectious brain damage depending on the types of infection (6), while 41% of patients may become epileptic in 10 years after an acute symptomatic seizure with SE (7).

While the AEDs are chosen in favor of acute control of SE, the potential short-term and long-term effects of such AEDs on SE-triggered epileptogenesis have been ignored in clinics. Since SE is highly epileptogenic, we hypothesized that AEDs that are used to control acute SE might affect the feasibility of the chronic development of epileptogenesis after SE. Therefore, to test this hypothesis, we designed experiments to compare the epileptogenic effects of SE that terminated different AEDs. In these experiments, an intraperitoneal (i.p.) injection of pilocarpine to a mouse produces a model of SE, which was terminated with three different AEDs, i.e., diazepam, midazolam, or phenobarbital. Then we evaluated the epileptogenic effects of SE with different AED treatments by examining SE-associated mortality, behavioral SRS, and pathological and cellular alterations of mice in the three different treatment groups.

## Results

### Pentobarbital Treatment Provides Less Neuroprotective Action Against Lethality in the Short Phase Following SE Induction

**Figure 1:**
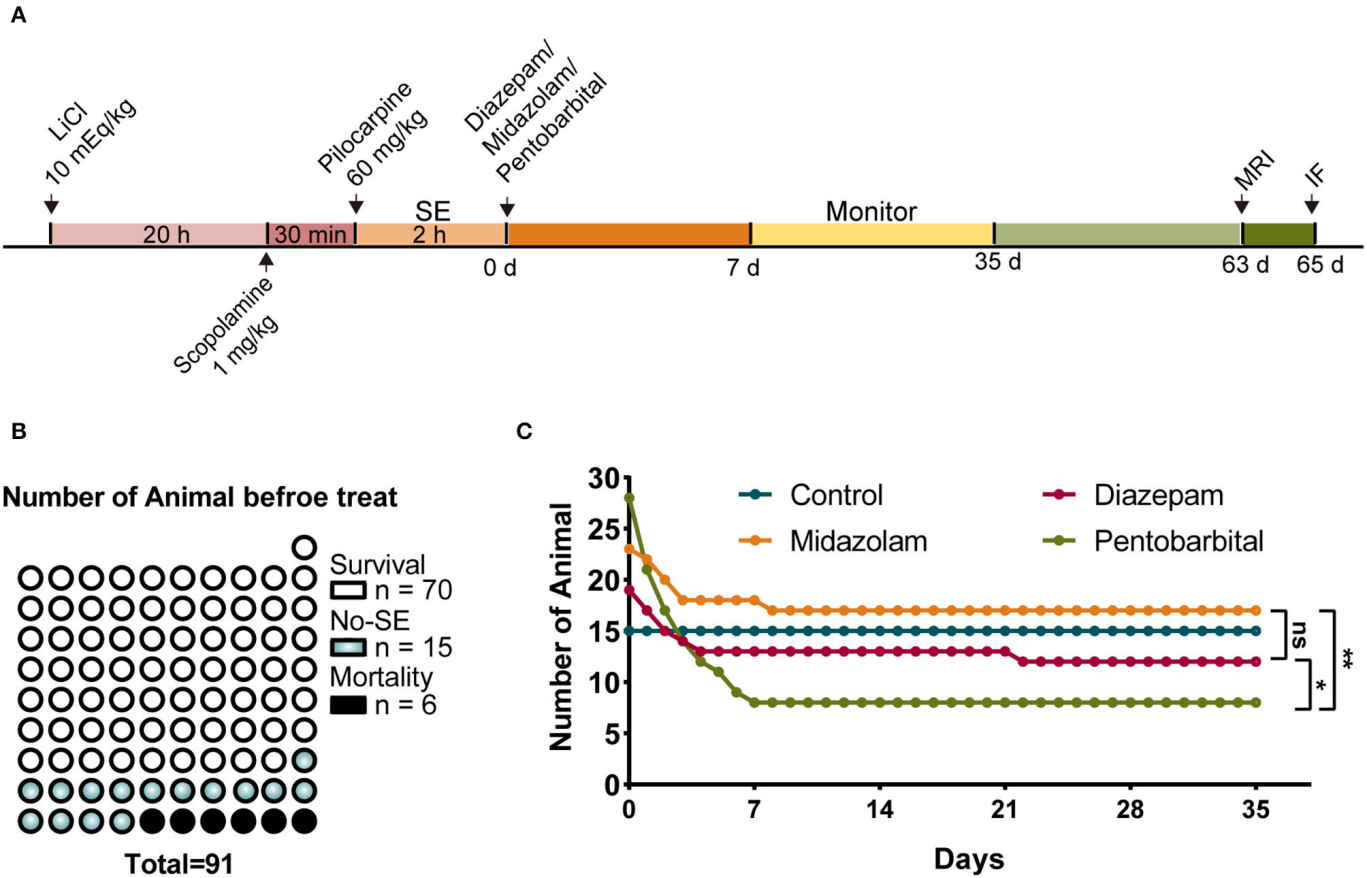
Comparison of mortality after status epilepticus (SE) treated with diazepam, midazolam, and pentobarbital. **(A)** Experimental paradigm of SE induction by LiCl-pilocarpine and a set of experiments performed after SE induction. (**B)** A number of mice of survival from SE, no SE responses (non-SE), and mortality after pilocarpine administration and before anti-SE treatments. **(C)** Number of mice allocated to the treatments with diazepam, midazolam, and pentobarbital to terminate SE and remaining within 5 weeks after SE. Control mice were no SE and received no treatment. The number of mice at the end of the fifth week/immediately after treatments: control group, n = 15/15; diazepam group, *n* = 12/19; midazolam group, *n* = 17/23; pentobarbital group, *n* = 8/28. Pentobarbital vs. diazepam, *p* = 0.0343; pentobarbital vs. midazolam, *p* = 0.0019; diazepam vs. midazolam, *p* = 0.5159; Fisher's exact test. *p < 0.05, **p < 0.01, ns indicates no significant difference.

Pilocarpine-induced SE is a well-established translatable animal model mimicking human SE in behavioral symptoms, pharmacological responses, and the development of SRS of temporal lobe epilepsy after a latent period (8, 9). Therefore, we used the pilocarpine model of SE and SRS to observe the short-term and long-term changes during epileptogenesis in mice in our experiments (Figure 1A). SE was induced with lithium chloride (LiCl)-primed pilocarpine (60 mg/kg i.p.). At the end of the induction of SE for 2 h, SE was terminated with an i.p. injection of one of the investigated AEDs, i.e., diazepam (5 mg/kg), midazolam (10 mg/kg), and pentobarbital (37.5 mg/kg). Following the termination of SE, the mice were kept in home cages and received intensive care for 1 week. Then the mice were subjected to a set of experiments that includes behavioral monitoring for SRS, structural investigation using MRI, cellular morphology using immunohistochemistry to discriminate the effects of diazepam, midazolam, and pentobarbital on SE-induced epileptogenesis (Figure 1A).

In total, 91 mice were treated with LiCl-pilocarpine (Figure 1A). We observed that 70 (76.9%) mice showed SE without death, 15 mice (16.5%) did not show SE, and 6 (6.6%) mice had died in 2 h following pilocarpine treatment (Figure 1B). This is consistent with our previous studies (10, 11). The survival mice with SE were randomly allocated into 3 groups that were i.p. injected with diazepam (*n* = 19), midazolam (*n* = 23), or pentobarbital (*n* = 28) to terminate SE. The mice that did not show SE received no treatment. After SE induction, the survival of mice was daily calculated for 5 weeks. We found that none of the mice without SE (*n* = 15) died in the 5 weeks. While, in the treatment groups, the death of mice had occurred almost entirely in the first week after SE induction, reflecting the SE-associated but not SRS-associated short-term effects of treatments. Among the three treatment groups, the mice in the pentobarbital group had the highest mortality (Figure 1C; pentobarbital vs. diazepam, *p* = 0.0343; pentobarbital vs. midazolam, *p* = 0.0019; diazepam vs. midazolam, *p* = 0.5159; Fisher's exact test). Therefore, SE treated with pentobarbital provides less neuroprotective action in the short phase following SE induction.

### Midazolam Treatment Provides Relative Long-Term Benefit Against the Development of SRS

**Figure 2:**
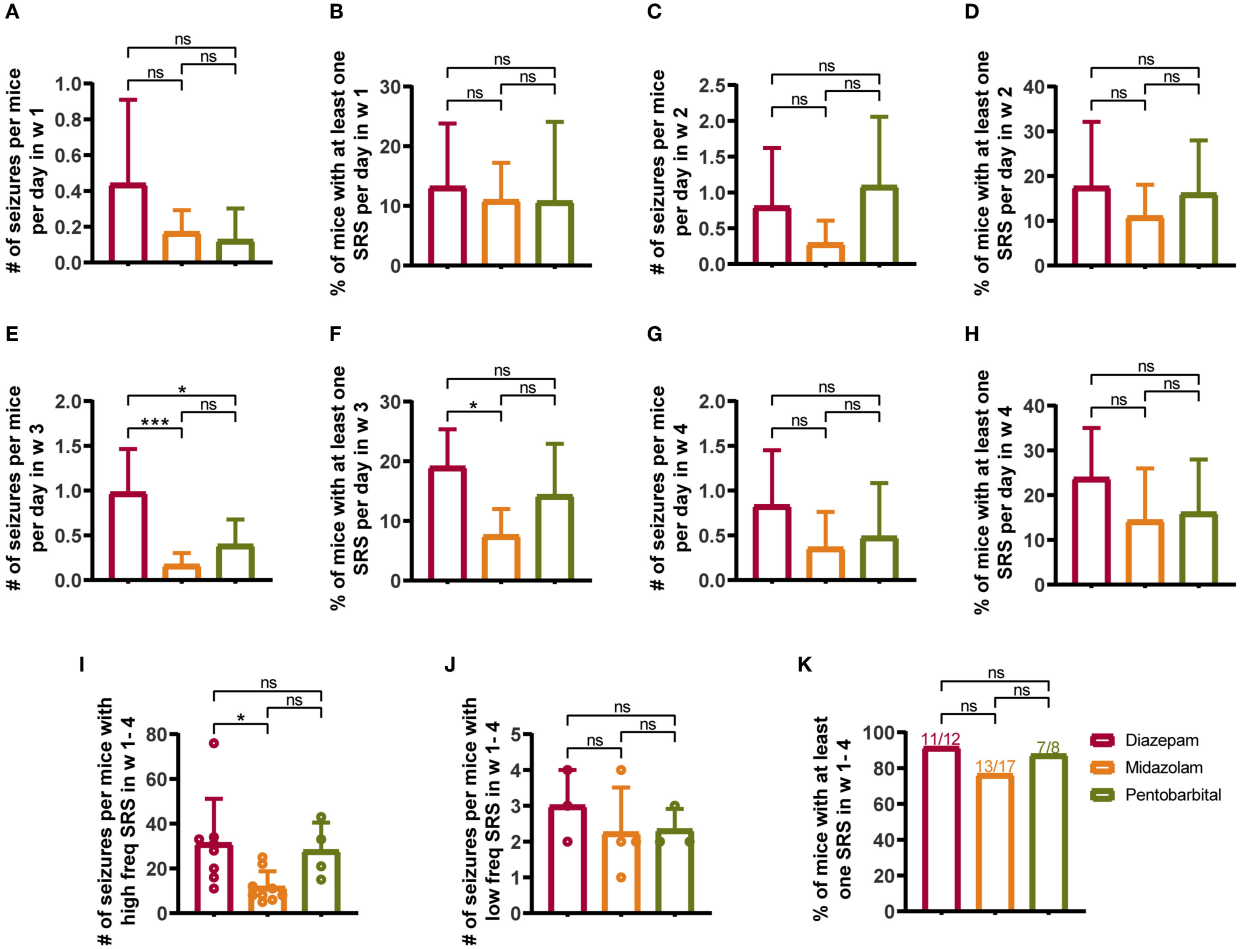
Comparison of spontaneous recurrent seizures (SRSs) monitored for 4 weeks (W 1–4) from 7th to 35th days after SE treated with diazepam, midazolam, and pentobarbital. **(A)** Number of seizures per mouse per day in week 1 of different treatment groups. **(B)** Percentage of mice with at least one SRS per day in week 1 of different treatment groups. **(C)** Number of seizures per mouse per day in week 2. **(D)** Percentage of mice with at least one SRS per day in week 2. **(E)** A number of seizures per mouse per day in week 3. **(F)** Percentage of mice with at least one SRS per day in week 3. **(G)** Number of seizures per mouse per day in week 4. **(H)** Percentage of mice with at least one SRS per day in week 4. **(I)** A number of seizures per mouse with high-frequency SRS which was defined that the total number of seizures of a mouse was more than 4 in a week. **(J)** Number of seizures per mouse with low-frequency SRS, which was defined that the total number of seizures of a mouse was 4 or less per week. **(K)** Percentage of mice with at least one SRS in weeks 1–4. **(A–J)** One-way ANOVA with *post-hoc* Tukey's multiple comparisons test. **(K)** Fisher's exact test, *p < 0.05, ***p < 0.001, ns indicates no significant difference. Data were presented as mean ± SD. See also Supplementary Table 1.

From the seventh day after SE induction, the mice were subjected to video monitoring for 4 weeks (w 1–4) to allow the detection of chronic SRS (Figure 1A). To reduce the contamination of normal vs. epileptic behaviors, only severe seizures scored > 3 according to the Racine scale (12) were included in the statistical calculation. Weekly statistics showed that midazolam treatment led to relatively milder SRS, as in the third week (Figures 2E,F), but not in the first 2 weeks and the last week of seizure monitoring (Figures 2A–H and Supplementary Table S1), both the number of seizures per mice per day (Figure 2E; diazepam 0.9762 ± 0.4876, midazolam 0.1681 ± 0.1334, *p* = 0.0007) and the percentage of mice with at least one seizure per day (Figure 2F; diazepam 19.05 ± 6.3%, midazolam 7.56 ± 4.445%, *p* = 0.0126) were significantly lower in the midazolam group comparing with the diazepam group. Meanwhile, the relatively milder severity of SRS in the midazolam group was illustrated by the total number of seizures in 4 weeks of seizure monitoring showing a lower occurrence of seizures per mice with high-frequency SRS (Figure 2I; diazepam 31.13 ± 19.97, midazolam 11.67 ± 7.089, *p* = 0.0189), but not low-frequency SRS (Figure 2J), comparing with the diazepam group. However, the percentage of mice with at least one SRS in weeks 1–4 of seizure monitoring was similar between the midazolam and diazepam groups. While pentobarbital treatment also led to the lower number of seizures per mice per day in week 3 (Figure 2E; diazepam 0.9762 ± 0.4876, pentobarbital 0.3929 ± 0.2835, *p* = 0.0115), overall, it had similar severity of SRS when compared with diazepam treatment (Figures 2A–K and Supplementary Table S1). These data suggest that midazolam treatment provides relative long-term benefits against the development of SRS.

### Midazolam Treatment Causes Less Hippocampal Sclerotic Atrophy

**Figure 3:**
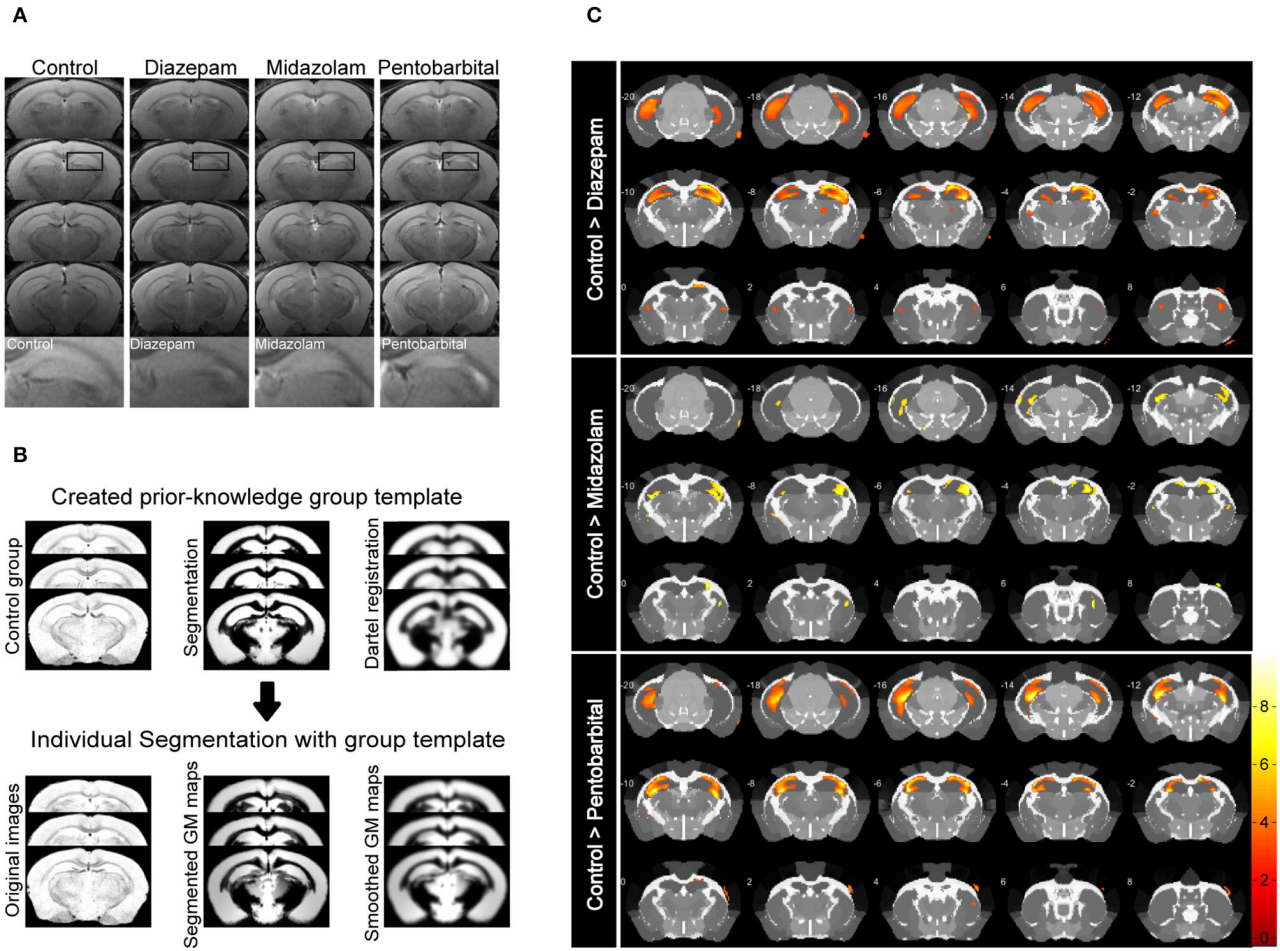
Voxel-based morphometry (VBM) analysis of MRI T2-weighted images showing differential hippocampal atrophy 9 weeks after SE received different treatments. **(A)** Hippocampal T2 maps along the rostro-caudal axis (up-down) showing sclerotic atrophy of hippocampus of control (non-SE and no treatment), diazepam, midazolam, and pentobarbital groups of mice after SE. The images in the lowest row are the amplifications of areas in the rectangle of the second row of images. **(B)** The template creation for spatial normalization of the segmented gray matter (GM) images and VBM procedure. **(C)** Each subfigure of the treatment group showed volumetric changes of GM when compared with corresponding subfigure of control group. Hippocampal GM densities in the midazolam group were significantly higher than other groups. The color scale bar on the right represents t-value of the statistical analysis. Control *n* = 6; diazepam *n* = 7; midazolam *n* = 7; and pentobarbital *n* = 4.

Magnetic resonance imaging (MRI) is able to reliably detect regional structural changes of the epileptic brain in relation to epileptogenesis of animal models (13, 14). Hippocampal atrophy is the most featured structural alternation of the pilocarpine-SE model of spontaneous chronic temporal epilepsy (13, 15, 16). To compare the long-term effects of diazepam, midazolam, and pentobarbital treatments antagonizing SE on brain structural changes, we performed MRI 9 weeks after SE induction. T2-weighted images showed that SE induction chronically causes hippocampal atrophy indicated by the shrinked hippocampal volume in all the treatment groups of mice when compared with the control group of mice that failed to be induced SE by pilocarpine (Figure 3A). However, voxel-based morphometry (VBM) analysis of T2-weighted images showed that midazolam treatment causes relatively less hippocampal sclerotic atrophy when compared to diazepam and pentobarbital treatments (Figures 3B,C and Table 1), suggesting that midazolam treatment against SE leads to less structural damages.

**Table 1:** Summary of Voxel-based morphometry (VBM) analysis.

**Regions**		**Control** **>** **Diazepam**		**Control** **>** **Midazolam**		**Control** **>** **Pentobarbital**	
**Full names of brain regions**	**Abbreviations of regions**	**k_**E**_**	**t**	**k_**E**_**	**t**	**k_**E**_**	**t**
Cornu ammonis, area 1	CA1	-	8.47	-	4.89	-	5.81
Cornu ammonis, area 2	CA2	-	-	3,630	5.4	-	-
Cornu ammonis, area 3	CA3	1,3927	10.05	1,272	4.34	12,546	10.28
Dentate gyrus, molecular layer	DG-mo	11,739	8.46	-	-	-	7.35
Dentate gyrus, polymorph layer	DG-po	-	5.09	-	-	-	-
Caudoputamen	CP	1,250	4.84	139	4.83	110	3.9
Alveus of hippocampus	ACAv2/3	132	4.37	-	-	-	-
Anterior cingulate area, ventral part, layer 2/3	ACAv5	186	4.57	-	-	-	-
Anterior cingulate area, ventral part, layer 5	alv	-	-	148	4.25	-	-
Anterior olfactory nucleus	AON	82	4.27	67	4.58	-	-
Ansiform lobule Crus 1	ANcr1	73	3.88	-	-	-	-
Arbor vitae	arb	-	-	46	4.04	-	-
Bed nuclei of the stria terminalis	BST	150	4.65	-	-	-	-
Cingulum bundle	cing	-	-	509	4.22	-	-
Genu of corpus callosum	ccg	32	3.47	35	3.97	-	-
Corpus callosum, body	ccb	-	-	157	3.94	-	6.8
Entorhinal area, medial part, dorsal zone, layer 1	ENTm1	57	3.8	119	4.76	-	-
Entorhinal area, lateral part, layer 2	ENTI2/3	-	-	57	3.55	-	-
Flocculus	FL	61	3.99	-	-	-	-
Internal capsule	int	608	4.65	-	-	-	-
Lateral septal nucleus, rostral (rostroventral) part	LSr	59	3.54	-	-	-	-
Lateral hypothalamic area	LHA	-	-	36	5.54	-	-
Primary motor area, Layer 6a	Mop6a	-	-	-	-	63	3.98
Orbital area, medial part, layer 1	ORBm1	581	7.61	-	-	240	5.65
Optic tract	opt	31	3.38	33	3.94	-	-
Posterior complex of the thalamus	PO	224	4.03	-	-	-	-
Sensory root of the trigeminal nerve	sV	83	4.36	-	-	-	-
Primary somatosensory area, upper limb, layer 6a	SSp-bfd6a	-	-	-	-	486	5.8
Primary somatosensory area, barrel field, layer 6a	SSs6a	-	-	-	-	-	3.6
Supplemental somatosensory area, layer 6a	SSp-ul6a	239	4.17	283	4.26	-	-
Postpiriform transition area	TR	-	-	-	-	39	5.19

*K_E_: larger than 30 contiguous voxels*.

*t: maximum T value*.

*Control n = 6; diazepam n = 7; midazolam n = 7; pentobarbital n = 4*.

*p <0.005 uncorrected for multiple comparisons*.

### Midazolam Treatment Results in Milder Neuronal Loss and Gliosis

**Figure 4:**
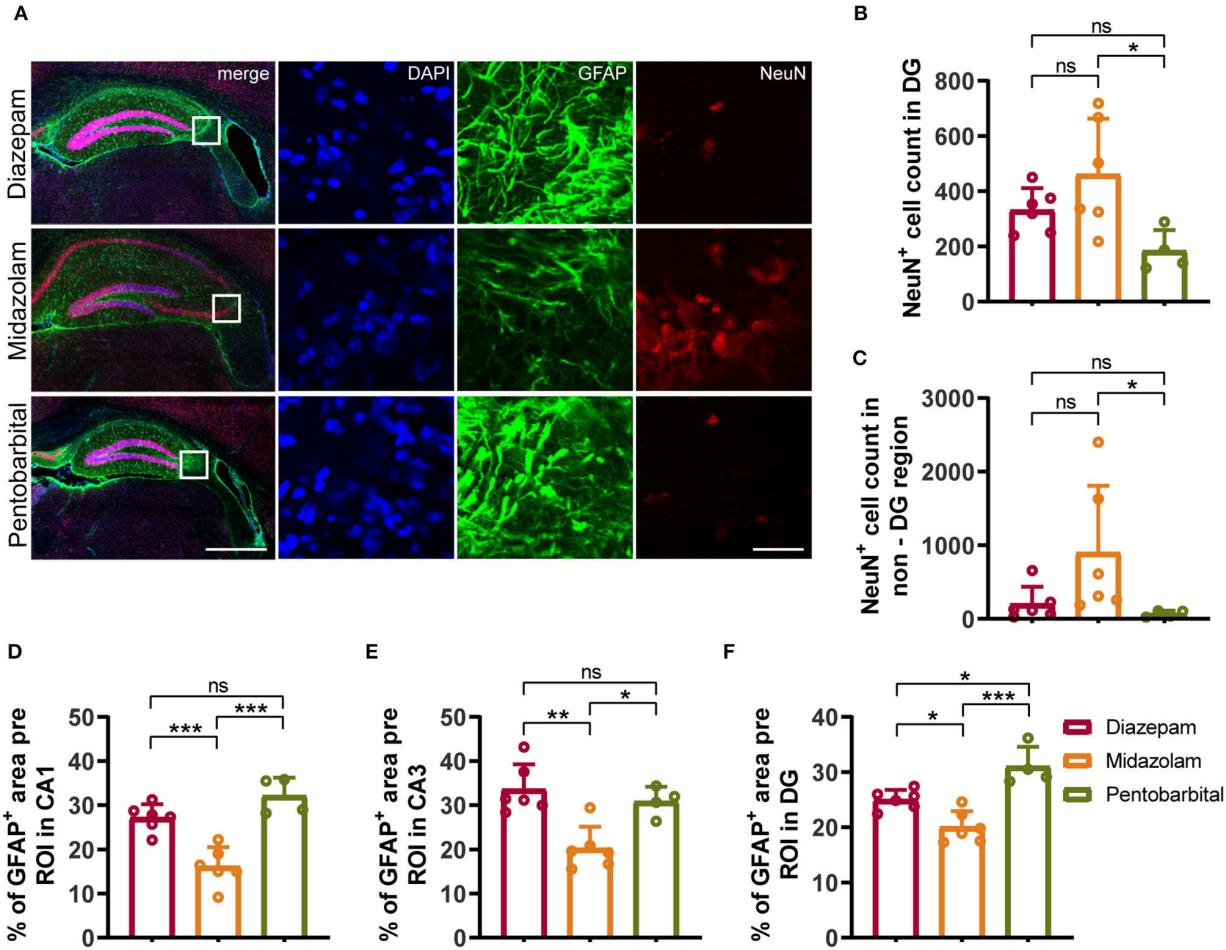
Immunohistochemical staining showed differential hippocampal neuronal loss and astrogliosis 9 weeks after SE received different treatments. **(A)** Representative staining for neurons (NeuN^+^) and astrocytes (GFAP^+^) of diazepam, midazolam and pentobarbital groups. Left scale bar: 500 μm; right scale bar: 100 μm. **(B)** Statistics of NeuN^+^ cell count in hippocampal DG area (diazepam, *n* = 6; midazolam, *n* = 6; pentobarbital, *n* = 4). **(C)** Statistics of NeuN^+^ cell count in hippocampal non-DG area (diazepam, *n* = 6; midazolam, *n* = 6; pentobarbital, *n* = 4). **(D)** Statistics of percentage of GFAP^+^ area per region of interest (ROI) in hippocampal CA1 area (diazepam, *n* = 6; midazolam, *n* = 6; pentobarbital, *n* = 4). **(E)** Statistics of percentage of GFAP^+^ area per region of interest (ROI) in hippocampal CA3 area (diazepam, *n* = 6; midazolam, *n* = 6; pentobarbital, *n* = 4). **(F)** Statistics of percentage of GFAP^+^ area per region of interest (ROI) in hippocampal DG area (diazepam, *n* = 6; midazolam, *n* = 6; pentobarbital, *n* = 4). One-way ANOVA with *post-hoc* Tukey's multiple comparisons test, *p < 0.05, **p < 0.01, ***p < 0.001, ns indicates no significant difference. Data were presented as mean ± SD.

**Figure 5:**
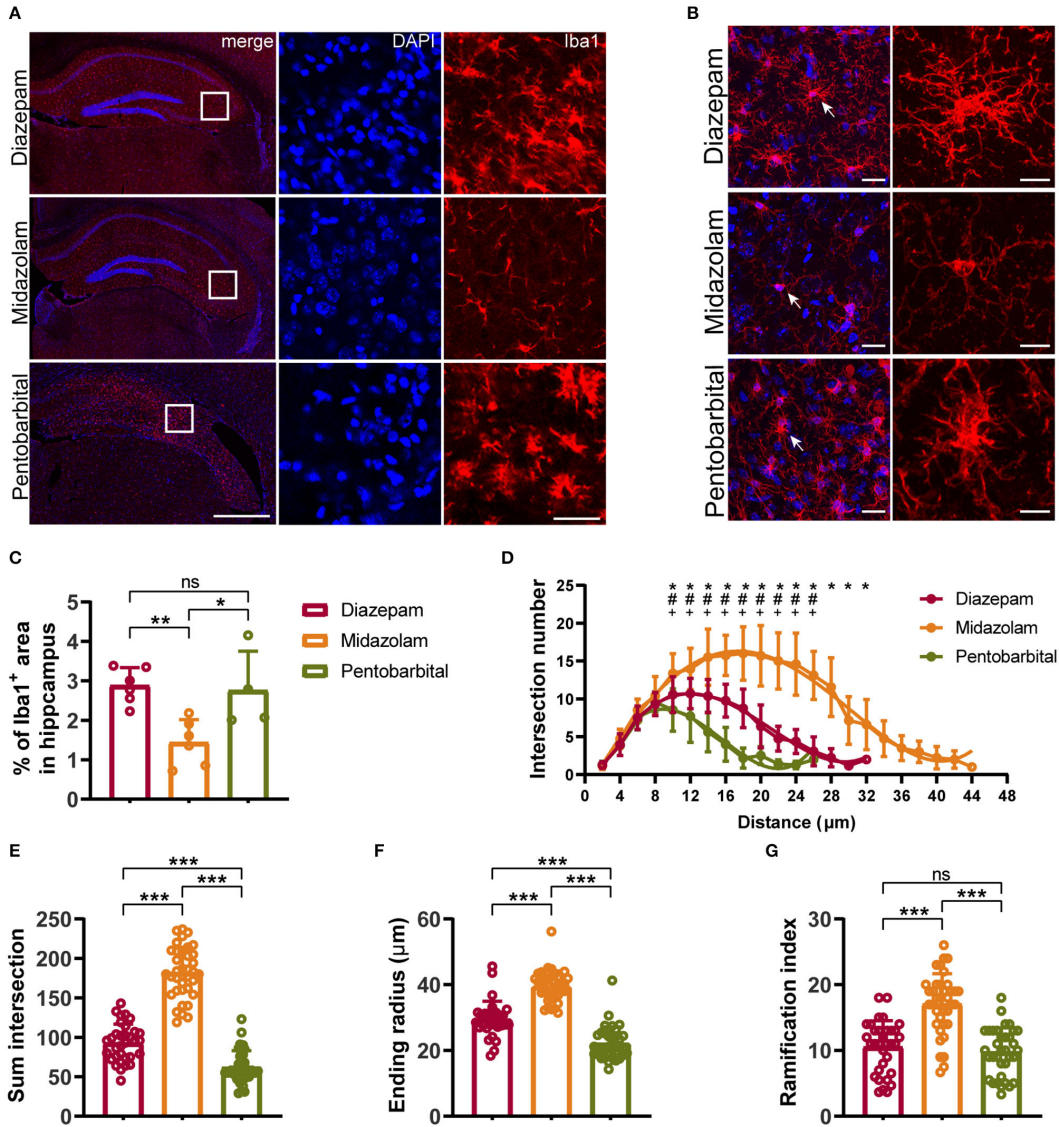
Immunohistochemical staining showing differential hippocampal microgliosis 9 weeks after SE received different treatments. **(A, B)** Representative staining for microglia (Iba1^+^) of diazepam, midazolam, and pentobarbital groups. Scale bar in (A): left, 500 μm; right, 100 μm. Scale bar in **(B)**: left, 20 μm; right, 10 μm. **(C)** Statistics of percentage of Iba1^+^ area in hippocampus. **(D)** Summary number of intersections as a function of the radial distance from soma by 2 μm increments showed that Iba1^+^ cells of midazolam group (*n* = 22) had greater numbers of ramifications than diazepam (*n* = 16) and pentobarbital (*n* = 21) groups at 10–38 μm distances from the soma. Diazepam vs. midazolam, *p < 0.05; diazepam vs. pentobarbital, #*p* < 0.05; midazolam vs. pentobarbital, +*p* < 0.05; two-way ANOVA with Tukey's multiple comparisons test. **(E)** Summary number of intersections per Iba1^+^ cell (diazepam, *n* = 33; midazolam, *n* = 37; pentobarbital, *n* = 35). **(F)** Length of maximum branch (ending radius) of Iba1^+^ cells (diazepam, *n* = 33; midazolam, *n* = 37; pentobarbital, *n* = 35). **(G)** Ramification index of Iba1^+^ cells (diazepam, *n* = 33; midazolam, *n* = 37; pentobarbital, *n* = 35). One-way ANOVA with *post-hoc* Tukey's multiple comparisons test, *p < 0.05, **p < 0.01, ***p < 0.001, ns indicate no significant difference. Data were presented as mean ± SD.

Neuronal death and reactive gliosis occurring soon after evoking SE and continuously during epileptogenesis contribute to the formation of brain tissue atrophy and progressive development of epilepsy (2, 17). Therefore, in the final experiments, we performed immunohistochemistry staining neuronal marker NeuN, astrocytic marker Glial Fibrillary Acidic Protein (GFAP), and microglia marker Iba1 to compare the chronic cellular alterations in the hippocampus of mice treated with diazepam, midazolam, and pentobarbital against SE. Consistent with the VBM analysis of MRI images, the hippocampal shapes shown by immunohistochemistry staining exhibit different severities of hippocampal sclerotic atrophy in different treatment groups. Overall, in the midazolam-treated mice, there is relatively less tissue sclerosis and neuronal loss so that cornu ammonis (CA) pyramidal cell layer can be observed in most of the samples. In contrast, in the diazepam- or pentobarbital-treated mice, the CA pyramidal cell layer is lost in most of the cases (Figures 4A, 5A).

NeuN^+^ quantification revealed that midazolam treatment resulted in a less neuronal loss as the number of NeuN^+^ counting was significantly higher when compared with pentobarbital treatment in the dantate gyrus (DG) (midazolam 461 ± 201.7, pentobarbital 184.5 ± 74.86, *p* = 0.0224) and non-DG regions (midazolam 899.2 ± 909.9, pentobarbital 68.38 ± 43.46, *p* = 0.0111; Figures 4A–C). Quantification of GFAP^+^ area revealed that midazolam treatment resulted in lower astrogliosis in the CA1, CA3, and DG when compared with both diazepam (CA1: diazepam 27.13 ± 3.077, midazolam 16.12 ± 4.38, *p* = 0.0007; CA3: diazepam 33.6 ± 5.63, midazolam 20.22 ± 4.895, *p* = 0.0011; DG: diazepam 25.01 ± 1.75, midazolam 20.06 ± 2.873, *p* = 0.0186.) and pentobarbital (CA1: pentobarbital 32.12 ± 4.085, *p* < 0.0001; CA3: pentobarbital 30.81 ± 3.366, *p* = 0.0136; DG: pentobarbital 31 ± 3.549, *p* < 0.0001) treatments (Figures 4A,D–F). Consistently, Quantification of Iba1^+^ area in hippocampus also revealed less gliosis in the midazolam treatment group when compared with both diazepam (diazepam 2.883 ± 0.4546, midazolam 1.439 ± 0.5788, *p* = 0.006) and pentobarbital (pentobarbital 2.755 ± 0.9972, *p* = 0.0222) treatment groups (Figures 5A,C). In line with this, Sholl analysis to evaluate the microglia architecture showed that midazolam treatment led to a less pro-inflammatory M1 phenotype as the number of intersections, ending radius, and ramification index of microglia processes were higher in the midazolam treatment group when compared with both diazepam and pentobarbital treatment groups (Figures 5B,D–G). These data indicate that mice with SE treated with midazolam result in milder neuronal loss and gliosis chronically following SE.

## Discussion

Although diazepam, midazolam, and pentobarbital are able to terminate SE effectively, their short-term and long-term consequences have not been compared. In the present study we demonstrate that, in the aspect of short-term consequence, the mice with SE treated with pentobarbital have a higher mortality than midazolam and phenobarbital. While in the aspect of long-term consequence, we demonstrate that mice with SE treated with midazolam showed less hippocampal sclerosis and gliosis and result in less development of SRS.

Diazepam and midazolam belong to benzodiazepines, which are a class of drugs widely used as sedatives, anticonvulsants, hypnotics, muscle relaxants, and anesthetics. The actions of benzodiazepines were fulfilled by targeting GABA_A_ receptors. When binding to the benzodiazepine side of GABA_A_ receptors, benzodiazepines do not directly cause functional opening of the receptors, but they allosterically modulate the receptors and promote their affinity for agonist GABA (18, 19). By this means, benzodiazepines enhance neuronal inhibition and reduce neuronal excitability (20–22). However, the exact mechanisms of modulation of GABA_A_ receptors by benzodiazepines are complicated, as there are many types of GABA_A_ receptors with different binding affinity for GABA and benzodiazepine. This is in that each GABA_A_ receptor comprises 5 subunits and there are up to 19 different subunits of the GABA_A_ receptor (23, 24). Therefore, even for the same type of GABA_A_ receptor, benzodiazepines (e.g., diazepam) may produce biphasic actions *via* separable subunit-dependent mechanisms (25). By this means, diazepam and midazolam may produce different effects on neuronal degeneration, gliosis, and neuroinflammation *via* different GABA_A_ receptors expressed by different cell types, thus leading to different effects on epileptogenesis.

Pentobarbital belongs to barbiturates, which are primarily prescribed as anticonvulsants and sometimes used as sedatives, hypnotics, and anesthetics. Pentobarbital can not only allosterically modulate GABA_A_ receptors (26) but also directly activate GABA_A_ receptors and induce GABAergic chloride currents (27). Additionally, pentobarbital is able to exert inhibition by suppressing some glutamate receptors and inhibiting glutamate release (26, 28, 29). Despite versatile inhibitory actions, barbiturates have the drawback of acute toxicity and lethality (26, 30). In line with this drawback, our data reveal that the mice with SE treated with pentobarbital have a higher mortality in the first week after SE induction when compared with midazolam and diazepam treatments. Such an acute neurotoxicity is likely to turn into chronic detriment *via* neuronal death, neuronal regeneration, gliosis, neuroinflammation, and so on. In terms of chronic epileptogenic effects of SE, although treatment with pentobarbital is followed by more neuronal loss, gliosis, and hippocampal sclerosis when compared with midazolam treatment, the statistics of SRS of mice show no significant difference between the pentobarbital and midazolam treatment groups. Presumably, this is due to insufficient animal numbers caused by SE-associated higher mortality of mice pentobarbital group.

In summary, by comparisons of epileptogenic effects of SE treated with diazepam, midazolam, and pentobarbital in the mouse LiCl-pilocarpine model of epilepsy, our data reveal an obvious advantage of midazolam vs. diazepam or pentobarbital in protecting the brain from epileptogenesis. LiCl-pilocarpine-induced murine SE represents the most well-established, clinically translatable SE model mimicking acute behavioral, electrographic, pharmacological, and pathological features and chronic epileptogenesis observed in human SE (31). Therefore, if midazolam provides as strong action to quench SE as diazepam, pentobarbital, and other AEDs in clinics, midazolam should be the first choice of anti-SE AEDs as it provides additional benefits against epileptogenesis. However, the molecular mechanisms underlying this intriguing long-term advantage and disadvantage of a single treatment with AEDs are yet to be studied in the future.

## Conclusions

Our data provide evidence that pentobarbital treatment for SE showed less neuroprotective action against lethality in the short phase following SE induction and that relatively to diazepam and pentobarbital, midazolam treatment for SE resulted in long-term benefits on epileptogenesis in that such SE causes less behavioral SRS, less hippocampal atrophy, and milder neuronal loss and gliosis. Therefore, in addition to the quality of acute control of SE, the short-term and long-term consequences of AEDs chosen to control SE should be taken into account in clinics. However, our study shall be followed by clinical studies regarding this concluding point and the studies relevant to the underlying molecular mechanisms.

## Methods

### Animals

Animals used in this study were purchased from Guangdong medical laboratory animal center, China. Animals were male ICR mice at 8–9 weeks of age and kept in a specific pathogen-free room with controlled temperature (22–24°C), humidity (50–75%), light conditions (12/12-h light-dark cycle, 08:00–20:00), and water and food *ad libitum*. Animals were treated according to the guidelines for Ethical Review of Animal Experiments at Southern Medical University in China, and the study was approved by the Southern Medical University Animal Ethics Committee.

### LiCl-Pilocarpine Model of Epilepsy and Evaluation of Seizures

Mice were intraperitoneally (i.p.) injected with LiCl (10 mEq/kg, Sigma #213233) 20 h prior to i.p. injection of pilocarpine (60 mg/kg, MedChemExpress #HY-B0726). To reduce peripheral effects of pilocarpine, mice were given an i.p. injection of scopolamine methyl nitrate (1 mg/kg, Sigma #S2250) 30 min before pilocarpine injection. Behavioral seizure severity in response to pilocarpine was assessed by Racine's standard classification into 0–6 stages (12). SE is defined as stages 4–6 epileptic seizures lasting for at least 30 min. Mice with SE received randomly i.p. injections of diazepam (5 mg/kg, Sanchine #H23021885), midazolam (10 mg/kg, Jiangsu Enhua #H10980025), or pentobarbital (37.5mg/kg, GENIA Biotech #P3760) at 2 h after pilocarpine treatment. From the seventh day after seizure induction, mice were videotaped 12 h (8 a.m. to 8 p.m.) every day for 28 days to capture SRSs. To avoid contamination of normal activities with mild seizures (stages 1–2) of mice, only seizures scored higher than stage 3 are included in the statistics of SRS.

### Immunohistochemistry

Mice were killed under anesthesia with pentobarbital sodium (75 mg/kg, i.p.). Brain slices were sectioned at 40 μm and were rinsed in phosphate-buffered saline (PBS). After washing the residual embedding medium (OCT), slices were blocked by 5% bovine serum albumin (BSA) with 1% Triton-X 100 for 1.5 h and then incubated with anti-Iba1 (1:800, Wako #019–19741), anti-NeuN (1:50, CST #24307), or anti-GFAP (GFAP; 1:800, Millipore #MAB360) in 5% BSA overnight at 4°C. After washing, slides were incubated with Alexa Fluor 488 (1:500, ZSGB-Bio #ZF-0511) or 594 (1:500, ZSGB-Bio #ZF-0513) goat anti-rabbit or goat anti-mouse secondary antibody for 1.5 h and then incubated with 4′,6-diamidino-2-phenylindole (DAPI; 1 μg/ml, Sigma #D9542) for 15 min at room temperature, washed 3 times with PBS for 15 min, and mounted onto slides. The slices were dried and mounted by coverslips with mounting medium. The samples were imaged using Nikon A1R confocal microscope. Cell counting and fluorescent area calculation were performed by ImageJ (version7). For the morphological analysis of microglia, confocal image stacks were collected using a 60 × oil-objective lens with a 0.5 μm interval. Branch analysis was performed by Sholl analysis with ImageJ. Microglial ramification index ramification was defined as the ratio between maximal intersections and the number of primary branches calculated when primary branches are valid and not zero.

### MRI Acquisition

A 7.0 T small animal MRI system (PharmaScan70/16 US, Bruker BioSpin MRI GmbH, Germany) was used to collect T2 data from the mice brain with ^1^H MRI CryoprobeTM 2 Element Array Kit for mice. The mice were anesthetized with 3% isoflurane before scanning and 1–1.5% isoflurane in oxygen during scanning. For T2 imaging, Bruker's Multi-Slice-Multi-Echo sequence was used. Scanning parameters were as follows: repetition time (TR) = 3577.557 ms, echo time (TE) = 35ms, total scan 23 slices, slice thickness = 0.5 mm, no slice gap, matrix = 256 × 256, field of view = 16 × 16 mm^2^, voxel size = 0.0625 × 0.0625 × 0.5 mm^3^, the scanning time was 3 min 48 s 963 ms. All the original Bruker images were converted to DICOM format with Paravision 6.0 software.

### VBM

For statistical parametric mapping (SPM)-voxel-based morphometry (VBM), structural data were analyzed using SPM 12 (https://www.fil.ion.ucl.ac.uk/spm/software/spm12/) running on MATLAB R2020a (MathWorks, Inc., Natick, MA, USA). T2-weighted images were manually re-oriented, placed the origin at the anterior commissure. Subsequently, the images were segmented to identify gray and white matter based on standard tissue probability maps (Turone Mouse Brain Template, https://www.nitrc.org/) using an old Segment for SPM. Exponentiated Lie Algebra (DARTEL) was used to estimate the deformations that best align the images together by iteratively registering the imported images with their average created a study-specific template for spatial normalization of the segmented gray matter (GM) images. The remaining subjects were registered non-linearly to this template using DARTEL existing template module and then normalized to Montreal Neurological Institute (MNI) space. Images were smoothed spatially with a full width at half maximum kernel of 6 mm. The results were visualized using xjView (https://www.alivelearn.net/xjview/). The TMBTA (Brain-Atlas) was used to define anatomic regions and the voxel of each region was reported. A group comparison of hippocampal GM was performed by using a *t*-test control minus the treatment group. The resulting t-statistic maps were thresholded at values of *p* < 0.005 (uncorrected). Only clusters larger than 30 and positive contiguous voxels were considered in the analysis.

## Data Availability Statement

The original contributions presented in the study are included in the article/Supplementary Material, further inquiries can be directed to the corresponding authors.

## Ethics Statement

The animal study was reviewed and approved by Southern Medical University Animal Ethics Committee.

## Author Contributions

YG and RC contributed to the experimental design. SL, SQ, BQ, YG, and RC established the methodology. XT performed the experiments and analyzed the data. ZZ, JZ, and SL helped to perform the experiments and maintain the animals. XT and RC wrote the manuscript. All authors contributed to the finalization and approved the content of the manuscript.

## Funding

This work was supported by a grant from the National Natural Science Foundation of China (NSFC) (31571035 and 31771127), the Natural Science Foundation of Guangdong Province (2016A030308005), and Guangdong Science and Technology Department (2018B030334001, 2018B030340001, and 2018B030335001).

## Conflict of Interest

The authors declare that the research was conducted in the absence of any commercial or financial relationships that could be construed as a potential conflict of interest.

## Publisher's Note

All claims expressed in this article are solely those of the authors and do not necessarily represent those of their affiliated organizations, or those of the publisher, the editors and the reviewers. Any product that may be evaluated in this article, or claim that may be made by its manufacturer, is not guaranteed or endorsed by the publisher.
