# Supplementary material for: A Comparison of Epileptogenic Effect of Status Epilepticus Treated With Diazepam, Midazolam, and Pentobarbital in the Mouse Pilocarpine Model of Epilepsy
Source: Front Neurol. 2022 May 20;13:821917. doi: 10.3389/fneur.2022.821917 (PMC9163813; doi:10.3389/fneur.2022.821917)
Supplement: Supplementary file 1 [file Table_1.pdf]

Supplementary Table 1. Weekly calculation of SRS.

	week	W1								W2							
	**day**	**1**	**2**	**3**	**4**	**5**	**6**	**7**	**per day**	**1**	**2**	**3**	**4**	**5**	**6**	**7**	**per day**
**Diazepam**	# of mice	13	13	13	13	13	13	13	/	13	13	13	13	13	13	13	/
	# of total seizure	0	8	17	4	1	1	9	/	12	2	0	11	21	27	0	/
	# of mice with at least one SRS	0	4	3	2	1	1	1	/	5	2	0	2	3	5	0	/
	total seizures / # of mice	0.00	0.62	1.31	0.31	0.08	0.08	0.69	**0.44**	0.92	0.15	0	0.85	1.62	2.08	0	**0.80**
	# of mice with at least one SRS / # of mice	0.00	0.31	0.23	0.15	0.08	0.08	0.08	**0.13**	0.38	0.15	0.00	0.15	0.23	0.38	0.00	**0.19**
**Midazolam**	# of mice	18	17	17	17	17	17	17	/	17	17	17	17	17	17	17	/
	# of total seizure	0	6	3	5	2	3	1	/	8	3	16	2	3	1	1	/
	# of mice with at least one SRS	0	3	2	2	2	3	1	/	1	2	4	1	3	1	1	/
	total seizures / # of mice	0	0.35	0.18	0.29	0.11	0.18	0.06	**0.17**	0.47	0.18	0.94	0.12	0.18	0.06	0.06	**0.29**
	# of mice with at least one SRS / # of mice	0.00	0.18	0.12	0.12	0.12	0.18	0.06	**0.11**	0.06	0.12	0.24	0.06	0.18	0.06	0.06	**0.11**
**Pentobarbital**	# of mice	8	8	8	8	8	8	8	/	8	8	8	8	8	8	8	/
	# of total seizure	0	1	1	0	1	0	4	/	20	18	10	3	3	0	7	/
	# of mice with at least one SRS	0	1	1	0	1	0	3	/	3	2	1	1	1	0	1	/
	total seizures / # of mice	0.00	0.13	0.13	0.00	0.13	0.00	0.50	**0.13**	2.50	2.25	1.25	0.38	0.38	0.00	0.88	**1.09**
	# of mice with at least one SRS / # of mice	0.00	0.13	0.13	0.00	0.13	0.00	0.38	**0.11**	0.38	0.25	0.13	0.13	0.13	0.00	0.13	**0.16**

	week	W3								W4							
	**day**	**1**	**2**	**3**	**4**	**5**	**6**	**7**	**per day**	**1**	**2**	**3**	**4**	**5**	**6**	**7**	**per day**
**Diazepam**	# of mice	12	12	12	12	12	12	12	/	12	12	12	12	12	12	12	/
	# of total seizure	5	13	12	7	17	21	7	/	21	8	3	3	16	15	3	/
	# of mice with at least one SRS	2	2	1	3	3	3	2	/	3	3	2	2	5	3	1	/
	total seizures / # of mice	0.42	1.08	1.00	0.58	1.42	1.75	0.58	**0.98**	1.75	0.67	0.25	0.25	1.33	1.25	0.25	**0.82**
	# of mice with at least one SRS / # of mice	0.17	0.17	0.08	0.25	0.25	0.25	0.17	**0.19**	0.25	0.25	0.17	0.17	0.42	0.25	0.08	**0.22**
**Midazolam**	# of mice	17	17	17	17	17	17	17	/	17	17	17	17	17	17	17	/
	# of total seizure	3	7	2	1	3	4	0	/	2	8	18	12	1	0	2	/
	# of mice with at least one SRS	1	2	2	1	1	2	0	/	2	5	5	3	1	0	1	/
	total seizures / # of mice	0.18	0.41	0.12	0.06	0.18	0.24	0.00	**0.17**	0.12	0.47	1.06	0.71	0.06	0.00	0.12	**0.36**
	# of mice with at least one SRS / # of mice	0.06	0.12	0.12	0.06	0.06	0.12	0.00	**0.08**	0.12	0.29	0.29	0.18	0.06	0.00	0.06	**0.14**
**Pentobarbital**	# of mice	8	8	8	8	8	8	8	/	8	8	8	8	8	8	8	/
	# of total seizure	2	6	0	1	5	5	3	/	2	1	0	1	5	14	4	/
	# of mice with at least one SRS	1	2	0	1	2	1	1	/	1	1	0	1	1	3	2	/
	total seizures / # of mice	0.25	0.75	0.00	0.13	0.63	0.63	0.38	**0.39**	0.25	0.13	0.00	0.13	0.63	1.75	0.50	**0.48**
	# of mice with at least one SRS / # of mice	0.13	0.25	0.00	0.13	0.25	0.13	0.13	**0.14**	0.13	0.13	0.00	0.13	0.13	0.38	0.25	**0.16**
